# Impact of SARS-CoV-2 exposure history on the T cell and IgG response

**DOI:** 10.1016/j.xcrm.2022.100898

**Published:** 2022-12-22

**Authors:** Roanne Keeton, Marius B. Tincho, Akiko Suzuki, Ntombi Benede, Amkele Ngomti, Richard Baguma, Masego V. Chauke, Mathilda Mennen, Sango Skelem, Marguerite Adriaanse, Alba Grifoni, Daniela Weiskopf, Alessandro Sette, Linda-Gail Bekker, Glenda Gray, Ntobeko A.B. Ntusi, Wendy A. Burgers, Catherine Riou

**Affiliations:** 1 Institute of Infectious Disease and Molecular Medicine, University of Cape Town, Cape Town, South Africa; 2 Division of Medical Virology, Department of Pathology, University of Cape Town, Cape Town, South Africa; 3 Department of Medicine, University of Cape Town and Groote Schuur Hospital, Cape Town, South Africa; 4 Cape Heart Institute, Faculty of Health Sciences, University of Cape Town, Cape Town, South Africa; 5 South African Medical Research Council Extramural Unit on Intersection of Non-communicable Diseases and Infectious Diseases, University of Cape Town, Cape Town, South Africa; 6 Center for Infectious Disease and Vaccine Research, La Jolla Institute for Immunology, La Jolla, CA, USA; 7 Department of Medicine, Division of Infectious Diseases and Global Public Health, University of California, San Diego (UCSD), La Jolla, CA, USA; 8 Desmond Tutu HIV Centre, University of Cape Town, Cape Town, South Africa; 9 South African Medical Research Council, Cape Town, South Africa; 10 Wellcome Centre for Infectious Diseases Research in Africa, University of Cape Town, Cape Town, South Africa

**Keywords:** SARS-CoV-2, COVID-19, T cell response, IgG response, hybrid immunity, Ad26.COV2.S vaccine

## Abstract

Multiple severe acute respiratory syndrome coronavirus 2 (SARS-CoV-2) exposures, from infection or vaccination, can potently boost spike antibody responses. Less is known about the impact of repeated exposures on T cell responses. Here, we compare the prevalence and frequency of peripheral SARS-CoV-2-specific T cell and immunoglobulin G (IgG) responses in 190 individuals with complex SARS-CoV-2 exposure histories. As expected, an increasing number of SARS-CoV-2 spike exposures significantly enhances the magnitude of IgG responses, while repeated exposures improve the number of T cell responders but have less impact on SARS-CoV-2 spike-specific T cell frequencies in the circulation. Moreover, we find that the number and nature of exposures (rather than the order of infection and vaccination) shape the spike immune response, with spike-specific CD4 T cells displaying a greater polyfunctional potential following hybrid immunity compared with vaccination only. Characterizing adaptive immunity from an evolving viral and immunological landscape may inform vaccine strategies to elicit optimal immunity as the pandemic progress.

## Highlights

• Repeated SARS-CoV-2 spike exposures enhance IgG responses
• Repeated spike exposures do not enhance the frequency of spike T cell responses
• The proportion of T cell responders reaches a plateau after three exposures
• Hybrid immunity induces more polyfunctional spike CD4 cells than vaccination only

## Graphical abstract

**Figure:**
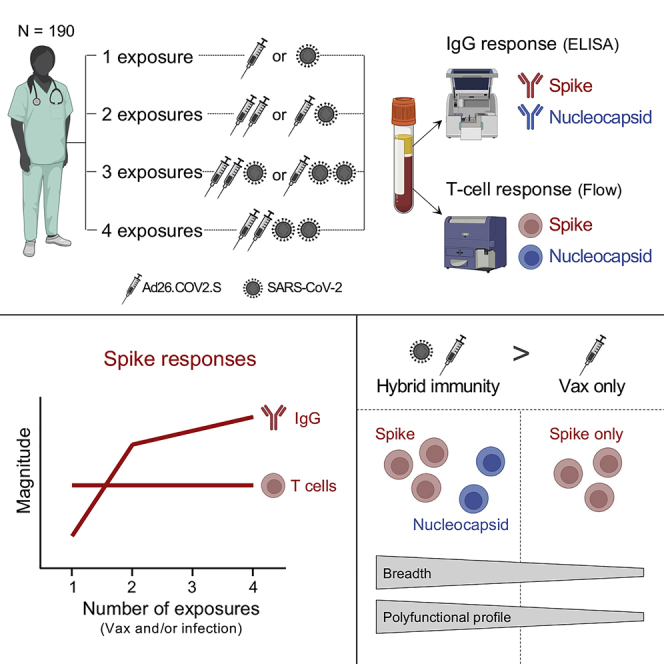

## Teaser

Keeton et al. investigate the effect of multiple exposures to SARS-CoV-2 spike (through infection and/or vaccination) on T cell and IgG responses. They show that an increasing number of exposures enhances the magnitude of IgG responses but has no major impact on the magnitude of SARS-CoV-2 spike-specific T cells.

## Introduction

It is now established that a coordinated and robust humoral and cellular response to severe acute respiratory syndrome coronavirus 2 (SARS-CoV-2) plays a key role in regulating infection, transmission, and disease severity.^1^^,^^2^ The COVID-19 pandemic over the past 2 and a half years has resulted in a complex virological and immunological landscape, with successive waves of distinct variants (from ancestral WU-1 to Omicron and its sub-lineages),^3^ the introduction of different vaccines and booster regimens,^4^ and subsequent emergence of breakthrough infections (BTIs). Hence, populations are now composed of heterogeneous groups, from rare immunologically naive individuals to those who have experienced multiple SARS-CoV-2 antigen exposures (through natural infection and/or vaccination), with diverse viral variants. It is thus essential to consider infection history when assessing the immune response to SARS-CoV-2. Recent publications assessing the impact of repeated SARS-CoV-2 exposures on antibody responses showed that an increased number of contacts with SARS-CoV-2 antigen(s) enhanced the quantity and quality of antibody responses, even against variants of concern.^5^^,^^6^^,^^7^^,^^8^^,^^9^ It is now clear that, compared with vaccination only, infection leads to the generation of a broader T cell response targeting the structural, non-structural, and accessory proteins of SARS-CoV-2, which are detectable in blood and at the sites of infection.^10^ These immunological properties could explain enhanced protection in the context of hybrid immunity when compared with vaccination alone.^11^ However, less is known about the effect of recurrent exposures on the T cell immune response, in particular the quantity, quality, and cross-reactivity of the response. In this study, to understand how the viral sequence of infection and/or vaccination, as well as repeated exposures, shape the immune response to SARS-CoV-2, we measured peripheral spike and nucleocapsid-specific T cell responses in individuals with a diverse SARS-CoV-2 exposure history, from a single exposure (i.e., one dose of Ad26.COV2.S vaccine or a COVID-19 episode) to four exposures (i.e., COVID-19 episode, prior to two Ad26.COV2.S doses, followed by a BTI), spanning four infection waves sequentially caused by the ancestral WU-1, Beta, Delta, and then Omicron variants.

## Results

### Study cohort

**Figure 1:**
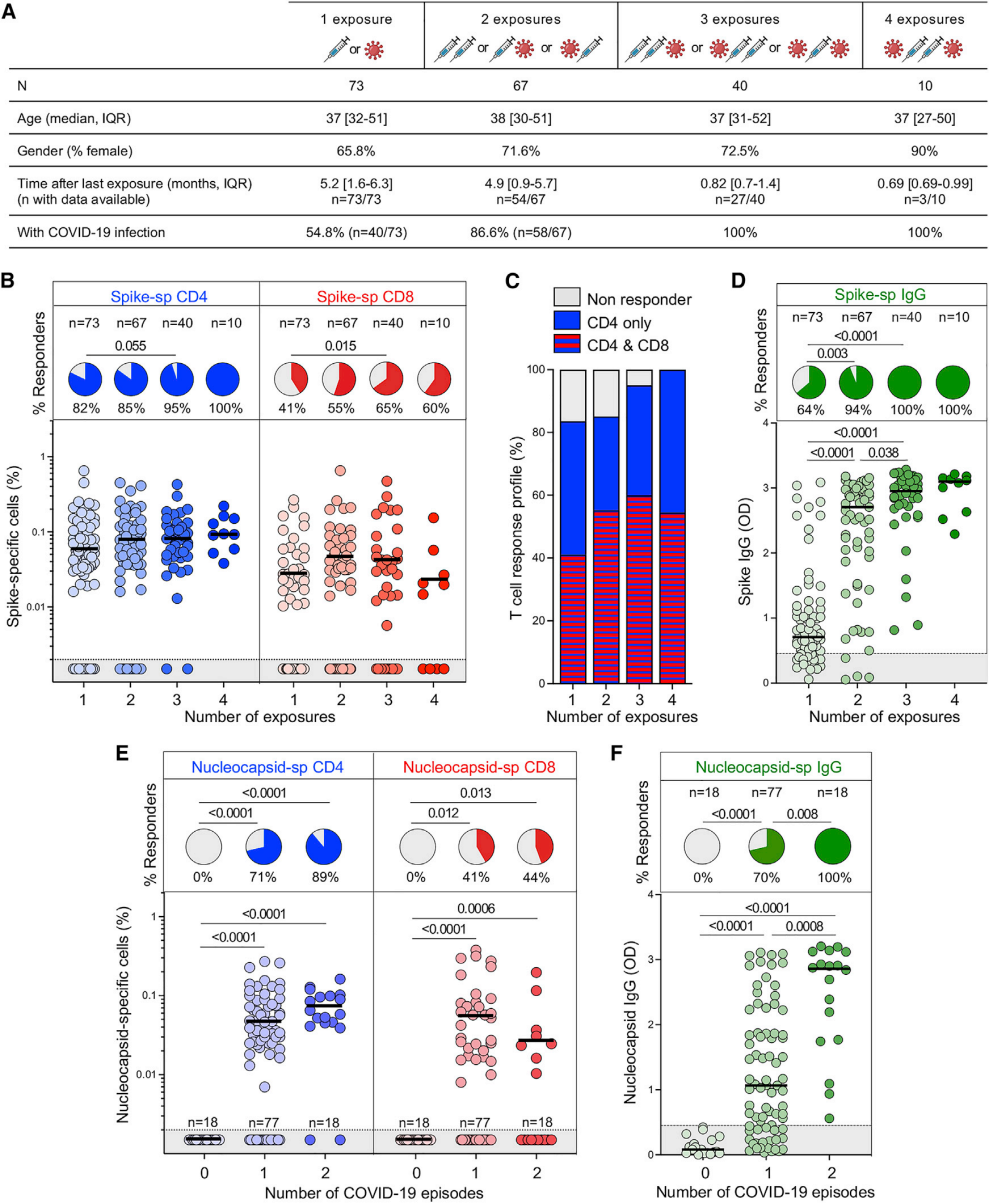
Comparison of SARS-CoV-2-specific T cell and IgG responses upon repeated exposures to SARS-CoV-2 antigens (A) Clinical characteristics of participants grouped according to their number of exposures. The syringe symbol corresponds to an Ad26.COV2.S vaccination. The virus symbol depicts SARS-CoV-2 infection. (B) Frequencies of ancestral SARS-CoV-2 spike-specific CD4^+^ (blue) and CD8^+^ (red) T cell responses (cells producing IFN-γ, TNF-α, or IL-2) in individuals with an increasing number of exposures. (C) Profile of the ancestral spike-specific T cell response based on the number of SARS-CoV-2 antigen exposures. (D) SARS-CoV-2 spike-specific IgG measured by ELISA in individuals with an increasing number of exposures. (E) Frequencies of the SARS-CoV-2 nucleocapsid-specific CD4^+^ (blue) and CD8^+^ (red) T cell responses (e.g., cells producing IFN-γ, TNF-α, or IL-2) in individuals with an increasing number of COVID-19 episodes. Horizontal lines indicate median values of responders. (F) SARS-CoV-2 nucleocapsid-specific IgG measured by ELISA in individuals with an increasing number of COVID-19 episodes. The number of participants included in each analysis is indicated on the graphs. Pie charts depict the proportion of participants exhibiting a detectable T cell response (blue for CD4^+^ T cells and red for CD8^+^ T cells). Statistical analysis was conducted using the Kruskal-Wallis test with Dunn’s multiple comparison test for the different exposure or episode groups. The proportion of responders is depicted by the pies on top of each graph. Chi-squared test was used to compare percentage of responders. Horizontal lines indicate median values of responders. See also Figures S1 and S2.

To define the impact of repeated antigen exposure on SARS-CoV-2 immunity, we measured T cell and immunoglublin G (IgG) responses in 190 healthcare workers from an ongoing longitudinal study,^4^^,^^12^ all of whom were offered the Janssen Ad26.COV2.S vaccine as part of the Sisonke vaccination study in South Africa. Participants were grouped according to their number of exposures to SARS-CoV-2 antigen through vaccination or a combination of infection and vaccination (Figure 1A). Infection was ascertained by a positive viral PCR test or conversion to nucleocapsid (N) seropositivity or, in the case of a BTI with an existing nucleocapsid antibody response from previous infection, >2-fold increase in anti-nucleocapsid IgG optical density (OD). The “1 exposure” group (n = 73) was composed of individuals vaccinated with a single dose of Ad26.CoV2.S (n = 33) or unvaccinated patients who experienced one COVID-19 episode (n = 40). The “2 exposures” group (n = 67) consisted of 9 individuals who received two doses of Ad26.COV2.S approximately 6 months apart, 36 participants who experienced a COVID-19 episode prior to receiving one dose of Ad26.COV2.S, and 22 participants who had a BTI after one dose of Ad26.COV2.S. The “3 exposures” group (n = 40) encompassed three different exposure profiles: 20 participants who experienced a COVID-19 episode prior to two doses of Ad26.COV2.S, 10 participants who had a BTI after two doses of Ad26.COV2.S, and 10 participants who had COVID-19 prior to one dose of Ad26.COV2.S followed by a BTI. Finally, the “4 exposures” group included 10 individuals who had COVID-19 prior to two doses of Ad26.COV2.S followed by a BTI. However, some asymptomatic infections could have occurred that we did not detect by nucleocapsid serology; BTIs have been described where nucleocapsid-specific IgG did not increase despite a positive PCR test.^13^ Thus, we cannot fully exclude the possibility that some participants may have experienced such an infection, where PCR testing was not performed, and nucleocapsid antibody levels did not change. Age and gender were comparable between each group. All COVID-19 cases were asymptomatic or mild and did not require hospitalization. The exact time since last exposure (infection or vaccination) was known for most participants, and the time range since the last negative sample was recorded for nucleocapsid seroconversions. The “1 exposure” and “2 exposures” groups had a median time since the last SARS-CoV-2 antigen exposure of 5.2 and 4.9 months, respectively. The majority of the “3 exposures” group were sampled <1 month since last exposure (0.81 months), and the “4 exposures” group all had samples collected <3 months after the last exposure, with 3/10 participants at a median 0.69 months.

### Evolution of SARS-CoV-2 T cell and IgG response upon repeated exposures to SARS-CoV-2 antigens

We first evaluated CD4^+^ and CD8^+^ T cell and IgG responses to ancestral SARS-CoV-2 spike according to the different number of exposures (SARS-CoV-2 infection and/or Ad26.COV2.S vaccination) (Figure 1A). As expected, abundant spike-specific CD4^+^ and CD8^+^ T cell responses were detectable after one to four antigen exposures (Figure 1B). The proportion of individuals exhibiting a detectable CD8^+^ T cell response to spike increased significantly between 1 and 3 exposures (p = 0.015), and the same trend was also observed for CD4^+^ T cell responders (p =0.055). Interestingly, no significant difference in the magnitude of spike-specific CD4^+^ or CD8^+^ T cells was detected regardless of the number of exposures. The evolution of the spike-specific T cell response upon repeated exposures is further illustrated in Figure 1C, showing that three exposures led to the highest proportion of concomitant CD4^+^ and CD8^+^ T cell responses. We then evaluated the polyfunctional potential of spike-specific T cells based on the number of exposures to SARS-CoV-2 antigen. With an increasing number of exposures, spike-specific CD4^+^ T cells were characterized by an increase in the proportion of cells producing interferon γ (IFN-γ) and interleukin-2 (IL-2) simultaneously, which was counterbalanced by a progressive decline in IL-2- and tumor necrosis factor α (TNF-α)-producing cells (Figure S1A). In contrast, the polyfunctional profile of spike-specific CD8^+^ T cells was not affected by repeated SARS-CoV-2 antigen exposures, with cells producing mainly IFN-γ alone (Figure S1B).

In parallel, we measured spike-specific serum IgG by ELISA (Figure 1D). The proportion of spike-specific IgG responders increased significantly between one and two exposures (64%–94%, respectively, p = 0.003), with a concomitant sharp increase in the magnitude of spike-specific IgG (median fold change: 3.8). A more modest but significant increase in magnitude was also observed between the second and third exposures (p = 0.038), with all participants seropositive by the third exposure. Of note, as previously reported,^14^^,^^15^ the frequency of spike-specific CD4^+^ T cells was associated with the magnitude of spike IgG (p < 0.0001, r = 0.41; Figure S2A). We also determined whether the magnitude of antibody or T cell responses were related to time since last exposure and found no association for spike-specific T cells or IgG in any exposure group (Figure S2B). Overall, these data show that spike-specific T cells and spike-specific IgG displayed distinct dynamics upon repeated SARS-CoV-2 antigen exposure and suggest that T cell and IgG responses plateau after three exposures.

Next, to define whether immune responses to SARS-CoV-2 nucleocapsid present a similar profile, we compared nucleocapsid-specific T cell and IgG responses in uninfected participants and patients who had one or two COVID-19 episodes (Figures 1E and 1F). T cells and IgG targeting nucleocapsid were undetectable in uninfected participants, as expected, and abundantly detectable in those who experienced infection. In convalescents, we did not detect any notable changes in the proportion of responders nor the magnitude of nucleocapsid-specific CD4^+^ and CD8^+^ T cells between individuals who experienced one or two COVID-19 episodes. The profile of the nucleocapsid-specific IgG response between one and two exposures was similar to that of spike-specific IgG, with a significant increase in both the proportion of responders (70%–100%, p = 0.008) and the magnitude of the response (median fold change: 2.7) (Figure 1F). Similarly, we found a positive association between the frequency of nucleocapsid-specific CD4^+^ T cells and nucleocapsid IgG (p < 0.0001, r = 0.52; Figure S2A).

**Figure 2:**
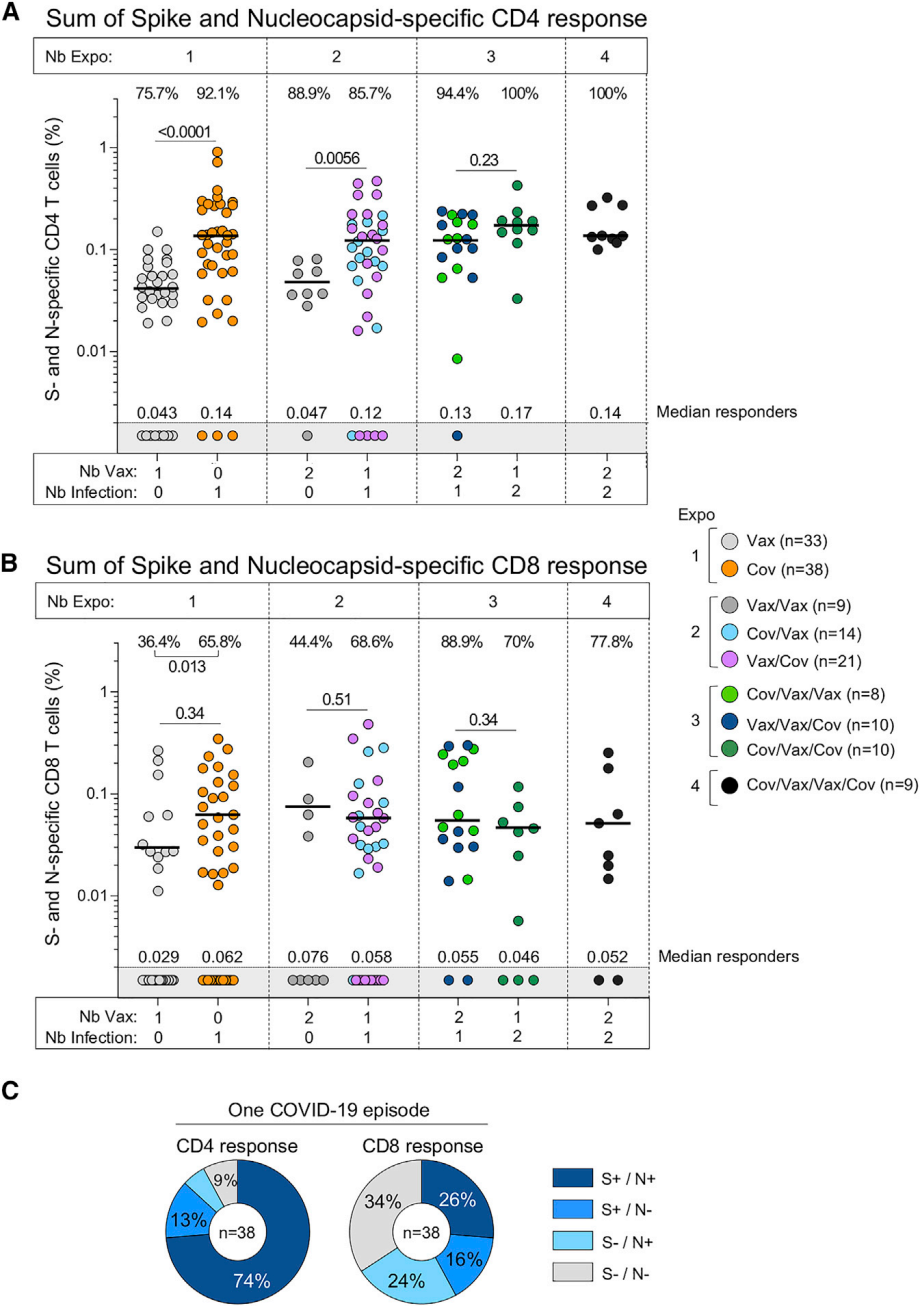
Profile of the combined spike- and nucleocapsid-specific T cell response upon different infection and/or vaccination exposures (A) Comparison of the combined frequency of spike- and nucleocapsid-specific CD4 T cells between groups. (B) Comparison of the combined frequency of spike- and nucleocapsid-specific CD8 T cells between groups. The proportion of responders is indicated at top of each graph. A chi-squared test was used to compare percentage of responders and a Wilcoxon unpaired t test to compare frequencies. Horizontal lines indicate median values of responders. Each color represents a different exposure profile. (C) Proportion of spike (S) and/or nucleocapsid (N) CD4 and CD8 responders after a SARS-CoV-2 infection. The number of participants in each sub-group is indicated inside each pie chart. See also Figure S3.

While we did not detect any significant change in the overall magnitude of the spike-specific T cell response upon repeated exposures, it is essential to acknowledge that infection leads to the generation of a broad T cell repertoire targeting SARS-CoV-2 structural, non-structural, and accessory proteins. Hence, in the context of infection or hybrid immunity, the overall magnitude of the T cell response to SARS-CoV-2 is expected to surpass that of individuals who have been vaccinated and have not been infected. To illustrate this, we compared the combined frequency of spike- and nucleocapsid-specific T cell responses upon different infection and/or vaccination exposures (Figure 2). As expected, in the context of infection or hybrid immunity, the frequency of spike- plus nucleocapsid-specific CD4 T cells was significantly higher than vaccination alone (Figure 2A). This superior CD4 response magnitude persisted even when compared with two doses of vaccine. Interestingly, for the CD8 response, the combined frequency of spike- and nucleocapsid-specific specific CD8 T cells was not significantly improved after infection or hybrid immunity compared with vaccination alone, but the proportion of responders was augmented (Figure 2B). Indeed, evaluating the proportion of spike and/or nucleocapsid responders after a SARS-CoV-2 infection showed that while two-thirds of the CD4 responders mounted a response to both spike and nucleocapsid, only a quarter of the CD8 responders exhibited a dual response and another quarter mounted a nucleocapsid response in the absence of a spike response (Figure 2C). The proportion of spike and nucleocapsid T cell responders stratified by their exposure history is presented in Figure S3. Thus, it is important to acknowledge that the overall magnitude of SARS-CoV-2-specific T cell response was clearly underestimated in our study as responses to other SARS-CoV-2 structural and accessory proteins were not considered.

### The type and order of exposures does not impact the profile of spike-specific T cell and IgG responses

To define whether a different order of infection and/or vaccination affects the profile of T cell and IgG responses, we compared spike-specific T cells and IgG responses in each group stratified by the sequence of exposures (vaccination versus infection for the “1 exposure” group; vaccination/vaccination, infection/vaccination, or vaccination/infection for the “2 exposures” group; and infection/vaccination/vaccination, vaccination/vaccination/infection, or vaccination/infection/vaccination for the “3 exposures” group). Clinical data for each sub-group are presented in Table S1. Where the date of infection was known or estimated within a narrow time window due to closely spaced study visits, we assigned these infections to the dominant circulating variant of that infection wave due to the highly virologically distinct infection waves that have occurred in South Africa.

**Figure 3:**
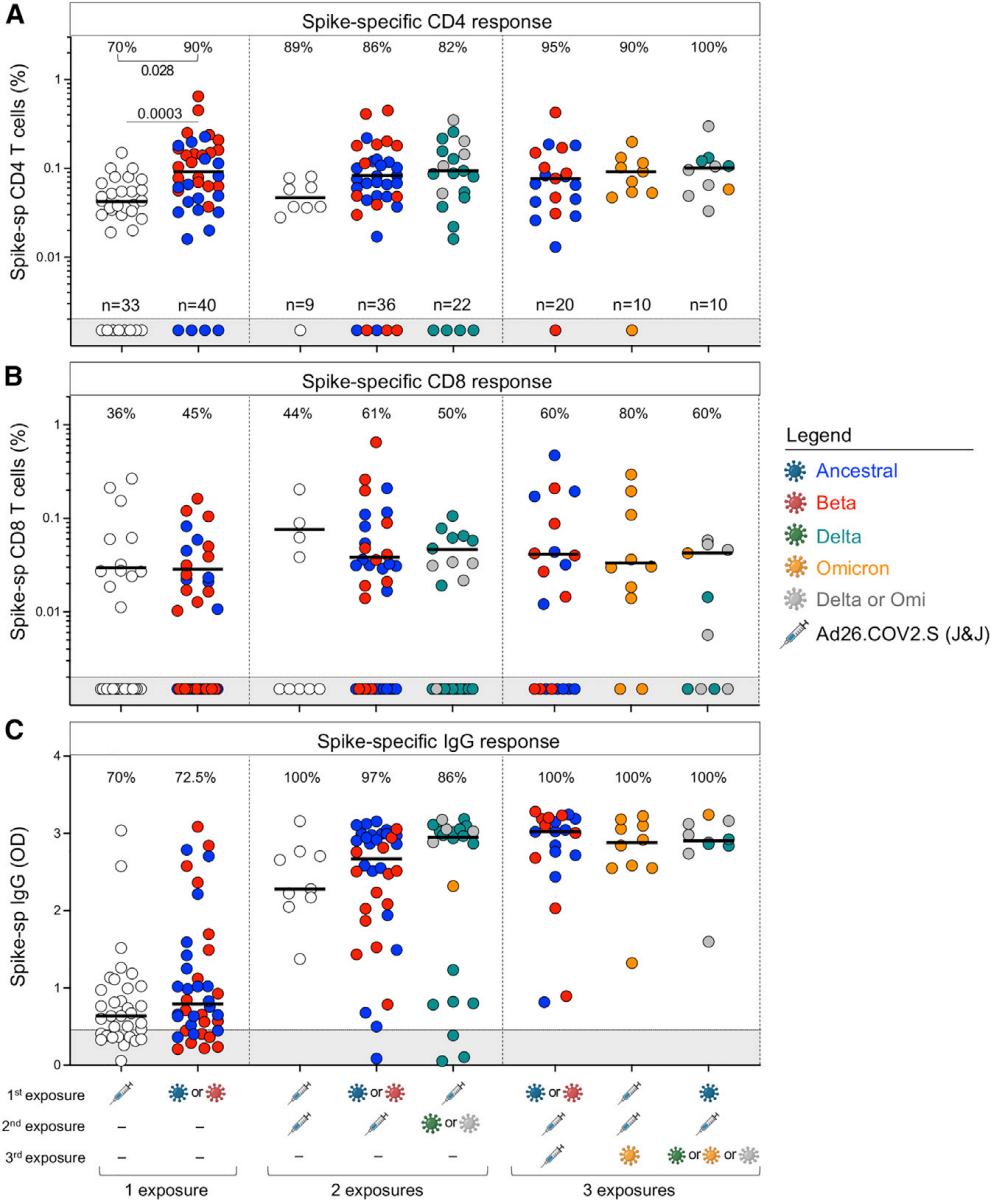
Comparison of SARS-CoV-2 spike-specific T cell responses in individuals with a different order of exposures (A) Comparison of the frequency of ancestral spike-specific CD4^+^ T cell responses. (B) Comparison of the frequency of ancestral spike-specific CD8^+^ T cell responses. (C) Comparison of ancestral spike-specific IgG responses. The sequence of exposure (vaccination or infection) for each group is indicated below the graph. Each SARS-CoV-2-infecting variant is depicted with a different color (see legend). For individuals infected twice, the color of the circle represents the breakthrough infection variant. For those infected with an unknown variant, this was either Delta or Omicron. The number of participants included in each group is indicated on the graphs. The proportion of participants exhibiting a detectable T cell response is indicated on top of each graph. Horizontal lines indicate median values of responders. Statistical comparisons were performed using a Kruskal-Wallis test with Dunn’s multiple comparison test or the chi-squared test to compare the percentage of responders. See also Table S1.

**Figure 4:**
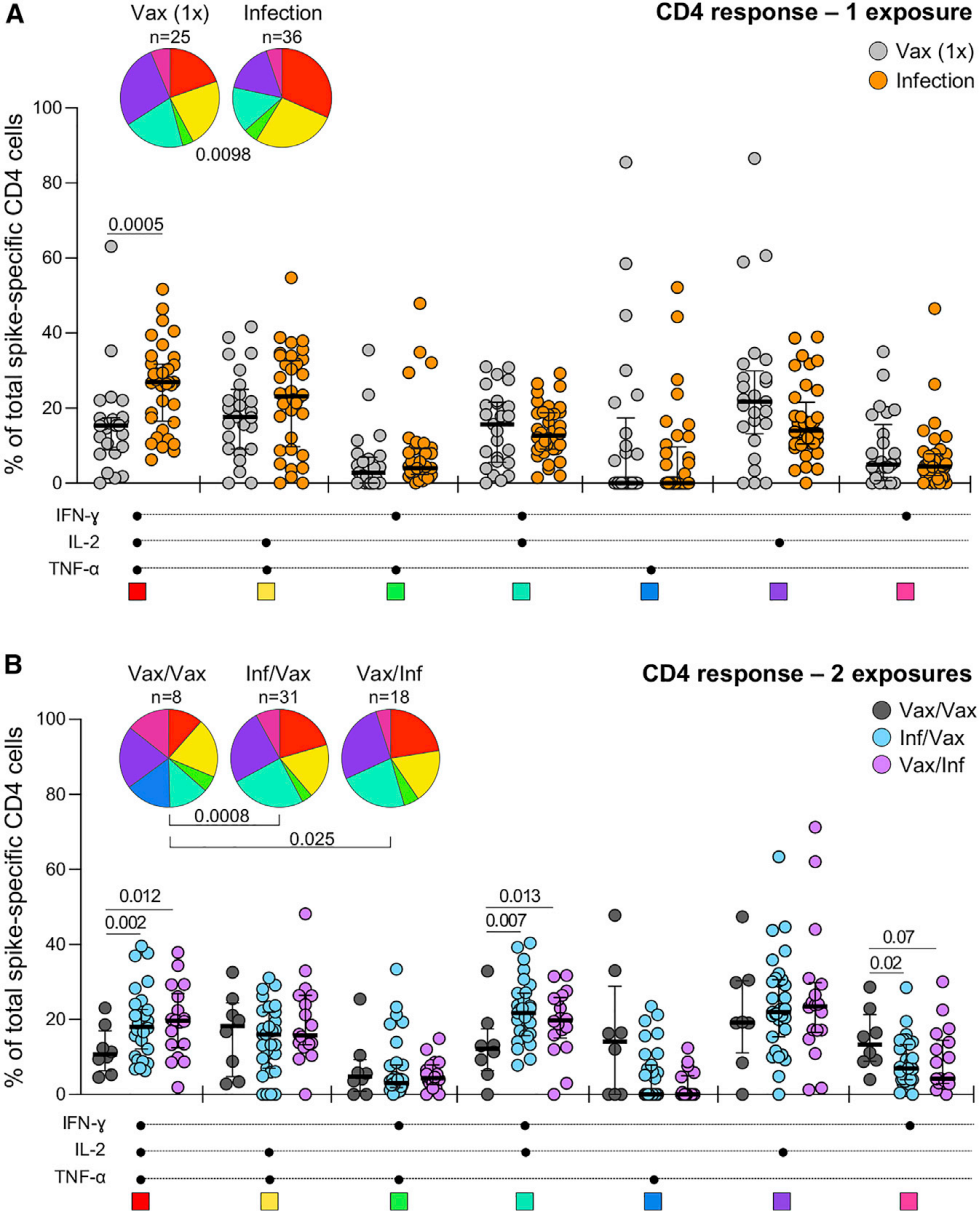
Polyfunctional profiles of spike-specific CD4^+^ T cells after one or two antigen exposures (A) Comparison of the polyfunctional profile of spike-specific CD4^+^ T cells after a single vaccination (gray circles) or one episode of infection (orange circles). (B) Comparison of the polyfunctional profile of spike-specific CD4^+^ T cells after two exposures, namely two vaccinations (dark gray circles), infection followed by a single vaccination (blue circles), or single vaccination followed by infection (purple circles). The median proportion and IQR are shown. Each response pattern (i.e., any possible combination of IFN-γ, IL-2, or TNF-α expression) is color coded, and data are summarized in the pie charts. Statistical comparisons were performed using a permutation test for the pie charts and a Wilcoxon unpaired t test for each response pattern. The number of participants included in each graph is indicated on top of the pie charts.

A significant difference in the spike-specific CD4^+^ T cell profile was only observed within the “1 exposure” group where both the proportion of responders (70% versus 90%, p = 0.028) and the frequency of spike-specific CD4^+^ T cell responses (p = 0.0003) was significantly higher in the SARS-CoV-2-infected group compared with the Ad26.COV2.S-vaccinated individuals (Figure 3A). On the contrary, spike-specific CD8^+^ T cell and IgG responses were comparable between these two sub-groups (Figures 3B and 3C). For the “2 exposures” and “3 exposures” groups, there was no significant difference in the proportion of responders and the magnitude of spike-specific T cells and IgG regardless of the sequence of exposures (Figures 3A–3C). Moreover, for participants who experienced a COVID-19 episode, the infecting strain did not appear to impact the profile of spike-specific T cell responses. When comparing the polyfunctional profile of spike-specific CD4^+^ T cells according to the nature and sequence of exposures, our data show that in the context of a single exposure, CD4 responses to natural infection were enriched in polyfunctional cells (producing IFN-γ, IL-2, and TNF-α) compared with those who received a single dose of Ad26.COV2.S (Figure 4A). Similarly, for persons who experienced two exposures, hybrid immunity (vaccination and infection, regardless of the order) induced a more polyfunctional profile compared with two doses of Ad26.COV2.S (Figure 4B).

Overall, these data show that (1) in the case of a single exposure, SARS-CoV-2 infection induces a more robust spike-specific CD4^+^ T cell response compared with a single dose of Ad26.COV2.S; (2) the number of SARS-CoV-2 exposures, rather than the sequence in which vaccination or infection occur, shapes the SARS-CoV-2 immune response; and (3) exposure to viral variants preserves T cell responses to ancestral spike.

### Profile and cross-reactivity of spike-specific T cells after BTIs

**Figure 5:**
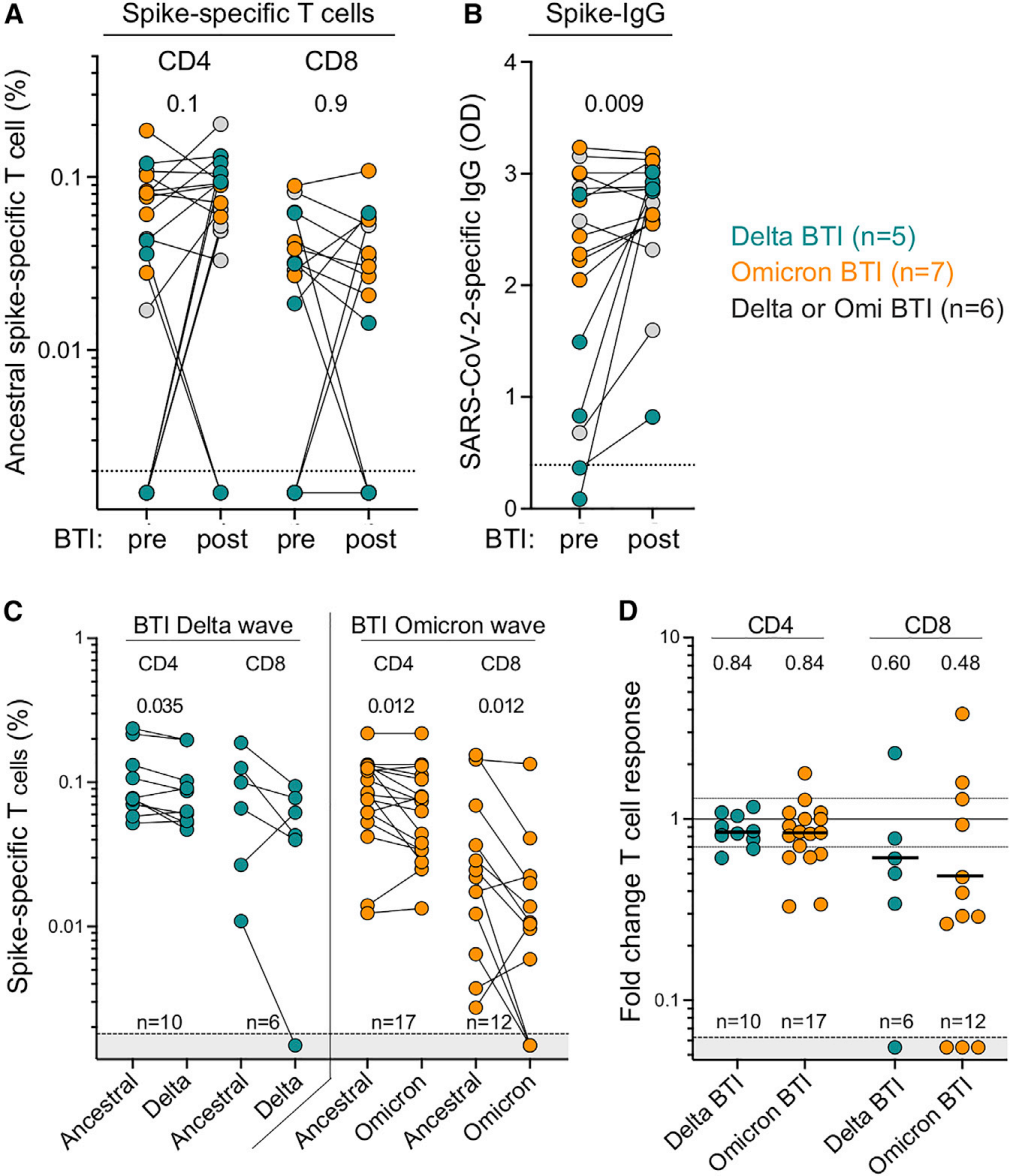
Impact of breakthrough infection on the magnitude and cross-reactivity of SARS-CoV-2 spike T cell responses (A) Frequencies of ancestral spike CD4^+^ and CD8^+^ T cell responses pre- and post-breakthrough infection (BTI) infection. (B) Magnitude of ancestral SARS-CoV-2 spike-specific IgG measured by ELISA pre- and post-BTI infection. (C) Cross-reactivity of spike CD4^+^ and CD8^+^ T cell responses after a Delta (left panel) or Omicron (right panel) BTI. (D) Fold change in the frequency of CD4^+^ and CD8^+^ T cells between ancestral and Delta spike responses (teal circles) and ancestral and Omicron responses (orange circles). Bars represent median fold change of responders. Delta BTIs are depicted by teal circles, Omicron BTI by an orange circle, and unknown variants (Delta or Omicron) by gray circles. The number of participants included in each graph is indicated, and the median value is indicated at the top of the graph. No significant differences were observed between CD4 or CD8 fold change using a Wilcoxon unpaired t test. For (A)–(C), a two-tailed Wilcoxon signed-rank test was used to assess statistical differences between paired samples. See also Table S2 and Figure S4.

For 18 participants who had a BTI with Delta or Omicron variants, we had access to longitudinal samples (pre- and post-BTI). The characteristics of each of these participants are presented in Table S2. Pre-BTI samples were obtained approximately 1 month after their last Ad26.COV2.S vaccination (median: 0.98 months, interquartile range [IQR]: 0.7–3.8), and post-BTI samples were taken a median of 4.2 months (IQR: 1.7–6) later. The exact date at which the BTI occurred was known for 4 participants who recorded a SARS-CoV-2-positive PCR test, while the remainder (n = 14) were characterized by nucleocapsid seroconversion or >2-fold increase in nucleocapsid IgG OD compared with the previous sample. Overall, in individuals experiencing a BTI, the median frequency of CD4^+^ or CD8^+^ T cells to ancestral spike was comparable pre- and post-BTI (Figure 5A), while spike-specific IgG increased significantly after BTI (p = 0.009; Figure 5B). These data are consistent with the results obtained from the cross-sectional cohort (Figures 1B and 1D). The evolution of T cell and IgG responses pre- and post-BTI for each individual participant is presented in Figure S4 according to their exposure history.

Next, we assessed the cross-reactive potential of spike-specific CD4^+^ and CD8^+^ T cells in samples collected after a Delta or Omicron BTI in an expanded set of participants with post-BTI samples available (n = 10 classified as Delta BTI and n = 17 as Omicron BA.1 BTI). CD4^+^ T cell frequencies to the BTI variant spike were significantly lower compared with ancestral spike (p = 0.035 for Delta and 0.012 for Omicron; Figure 5C), resulting in a median decrease in CD4 responses of 16% toward the BTI variant as demonstrated by fold change (Figure 5D). These data confirm the ability of CD4^+^ T cells to effectively cross-recognize SARS-CoV-2 variants^16^^,^^17^^,^^18^ even in highly mutated variants such as Omicron. For the CD8 response, the frequency of CD8^+^ T cells recognizing spike from the BTI variant was reduced by 50% or more in two-thirds of the participants (3/6 for Delta BTI and 8/12 for Omicron BTI) compared with the ancestral spike CD8 response (Figure 5D). This suggests that in some individuals who experienced a BTI, Delta or Omicron mutations may escape from specific HLA-restricted T cell responses induced by prior infection and vaccination.

## Discussion

Understanding the effects of repeated antigen exposures, through infection and/or vaccination, on the development of SARS-CoV-2 memory T cell and antibody responses is essential for determining susceptibility to subsequent infections and informing booster vaccination strategies. We compared SARS-CoV-2-specific T cell and IgG responses in individuals who experienced between one and four SARS-CoV-2 antigen exposures. When measuring spike-specific IgG, we found that the proportion of responders and magnitude of spike-specific IgG progressively increased upon repeated exposures. In contrast, the impact on spike-specific T cell responses appeared more modest, with robust responses at stable frequencies over repeated exposures, concomitant with an increased number of individuals mounting a T cell response.

Our results are in accordance with several publications showing that hybrid immunity and mRNA vaccination boosters are beneficial for the antibody response, enhancing its magnitude and broadening neutralizing potency even against highly divergent SARS-CoV-2 variants.^5^^,^^6^^,^^7^^,^^8^^,^^9^^,^^19^ However, humoral responses, generated upon natural infection or vaccination, can substantially and rapidly wane,^20^^,^^21^^,^^22^ emphasizing the importance of vaccination boosters.^23^ Less is known about the impact of repeated antigen exposures on T cell immunity toward SARS-CoV-2. First, primary SARS-CoV-2 infection or vaccination induces robust T cell immunity, with CD4 T cell responses being more prevalent than CD8 responses.^1^^,^^12^^,^^24^^,^^25^ Moreover, unlike antibodies, T cell responses are retained up to 6 to 12 months post-infection.^26^^,^^27^^,^^28^ In this study, we show that while a third exposure significantly increased the proportion of individuals exhibiting a T cell response to SARS-CoV-2 spike, the magnitude of those responses was not affected by repeated exposures. Since antigen-specific T cells were measured in the memory phase, it is possible that vaccination- or infection-induced T cell responses had reached an immunologic plateau with limited evolution even upon repeated stimulation. However, quantity may be contrasted with qualitative changes that occurred; we observed that increased exposures affected the polyfunctional profile of CD4^+^ T cells, resulting in a progressive increase in the proportion of IFN-γ^+^IL-2^+^ cells upon repeated exposures. This may arise through autocrine production of IL-2, which is thought to support memory T cell development by providing survival signals.^29^ Others have also described phenotypic characteristics changing after repeated exposures.^30^ Together, these observations demonstrate dynamic changes to SARS-CoV-2-specific T cells upon repeated exposures underlying stable response frequencies. However, while we did not observe any significant change in the overall frequency of peripheral T cells against spike upon repeated exposures, testing full-length spike could have obscured the generation of T cell specificities targeting additional spike epitopes, while other T cell specificities contracted. This could be done by assessing the T cell response against distinct sections of the spike protein, selectively covering the N-terminal domain, S1 chain, S2 chain, or the receptor-binding domain.^31^^,^^32^

Importantly, our study was limited to the analysis of spike- and nucleocapsid-specific T cell responses in the blood. There is now clear evidence that infection also stimulates mucosal immunity.^10^ SARS-CoV-2-specific resident T cells have been described in the nose and lungs of convalescent COVID-19 patients, and such responses were absent in individuals who had only been vaccinated.^33^^,^^34^ Moreover, a detailed comparison of the specificity of SARS-CoV-2 T cell responses between the nasal mucosa and peripheral blood showed that circulating SARS-CoV-2-specific T cell responses were dominated by spike-specific cells, while in the nasal mucosa, responses against nucleocapsid or NSP-12 were more prevalent.^33^ These data clearly indicate that, unlike vaccination, infection induces a broad T cell response targeting multiple SARS-CoV-2 proteins and promotes the establishment of SARS-CoV-2-specific tissue-resident T cells. It remains to be established whether vaccination after an infection has an impact on spike-specific T cell responses at sites of infection.

Although T cell responses generated upon vaccination or prior infection are highly cross-reactive with SARS-CoV-2 variants,^16^^,^^17^^,^^18^^,^^35^^,^^36^^,^^37^ we did not observe significant boosting of the T cell response after BTI. This suggests that BTIs may not lead to the generation of *de novo* T cells targeting mutated spike epitopes in variants. Indeed, when we measured cross-reactivity to Delta or Omicron spike after BTIs with these variants, CD4 responses were well preserved, but CD8 responses were less cross-reactive, as previously described for vaccinees and convalescent patients.^16^^,^^18^^,^^36^^,^^37^ We found that participants retained only 50% CD8 cross-reactivity to Omicron spike after BTIs during the Omicron surge. This may reflect antigenic imprinting, where the secondary response focuses epitope recognition on conserved spike epitopes. There is evidence for imprinting of the response for both T cell and B cell SARS-CoV-2 responses.^38^^,^^39^ For example, initial Beta and Omicron BA.4 infection led to greater cross-reactivity to multiple variants compared with Delta and Omicron BA.1 infections.^40^ However, our study was not designed to comprehensively assess the cross-reactive potential of SARS-CoV-2-specific T cells or identify the potential emergence of *de novo* SARS-CoV-2 T cell responses. To do so, it will be necessary to (1) measure the cross-reactivity of SARS-CoV-2 T cell responses to all SARS-CoV-2 variants of concerns both pre- and post-BTI and (2) probe T cell responses specifically targeting mutated epitopes from the breakthrough SARS-CoV-2 variant.

Whether exposure to specific variants shapes the T cell response to a similar extent remains to be fully defined for CD8^+^ T cell responses. So, too, it remains to be determined whether exposure history can be “rewritten” and expanded through heterologous vaccination^32^ or the use of adjuvants. It should be noted that BTI readily induces new non-spike T cell responses,^30^ which would supplement the cache of T cells available to engage and protect against severe disease upon viral re-encounter.

While we describe that the overall T cell response is stable (plateauing early after exposure through vaccination or infection), there is considerable inter-individual heterogeneity spanning 1.5 log in T cell frequencies to spike. Moreover, while all participants mounted spike-specific CD4^+^ T cell responses after four antigen exposures, approximately 20%–30% of participants remained refractory to the induction of spike-specific CD8^+^ T cell responses. Multiple studies demonstrate this response deficit for CD8^+^ T cell responses during vaccination and infection.^41^^,^^42^ Specific class I and II alleles have emerged as associating with increased or decreased spike or nucleocapsid-specific T cell responses.^43^^,^^44^ Which alleles are linked to CD8 hypo-responsiveness remains to be determined. There is evidence that the CD8 response is more narrowly directed than the considerable breadth of CD4 epitopes that are targeted (on average, 26–29 CD4 epitopes in spike^45^) and that the CD8 response is more affected by mutations in variants.^46^ These observations demonstrate the need to investigate SARS-CoV-2-specific CD8^+^ T cell responses in greater detail.

Overall, our study describes the quantity, quality, and dynamics of SARS-CoV-2-specific T cells in an increasingly complex immunological landscape. Multiple exposures result in limited immunological gains with respect to the magnitude of already robust virus-specific T cells in the circulation, but how boosting shapes T cell breadth, durability, and tissue homing remains an important question for further study. Differential exposure to distinct variants in different populations with diverse immunogenetics may affect future recognition of variants, and monitoring of vaccine efficacy and population immunity must continue as the pandemic rolls on.

### Limitations of the study

Our study had several limitations that should be considered. We did not measure neutralizing antibody responses. However, a host of studies have demonstrated that repeated exposures, including BTIs, improve neutralization titers and breadth against SARS-CoV-2 variants.^5^^,^^6^^,^^7^^,^^8^^,^^9^^,^^19^ While we show that the magnitude of the T cell response reaches a plateau after repeated SARS-CoV-2 exposures, it remains to be determined whether the breadth of the response is expanded by multiple exposures, which could be performed by epitope mapping studies and T cell receptor (TCR) clonotyping. We did not address durability of the T cell response since we measured responses only approximately 1 month after the third or fourth SARS-CoV-2 antigen exposure. It is plausible that multiple exposures may improve long term T cell memory^28^ and/or antigen-specific tissue resident T cells. In the context of hybrid immunity (particularly BTIs), SARS-CoV-2-specific T cells may migrate to and persist in the airways,^10^ leading to an underestimation of total T cell frequencies induced upon multiple exposures.

## STAR★Methods

### Key resources table

**Table:** 

REAGENT or RESOURCE	SOURCE	IDENTIFIER
**Antibodies**		

Purified NA/LE anti-human CD28 (clone 28.2)	BD Pharmingen	Cat# 555725; RRID:AB_2130052
Purified NA/LE anti-human CD49days (clone L25)	BD Pharmingen	Cat# 555501; RRID:AB_396068
LIVE/DEAD™ Fixable VIVID Stain	Thermo-Fisher	Cat# L34955
CD14 Pac Blue (clone TuK4)	Thermo-Fisher	Cat# MHCD1428; RRID:AB_10373537
CD19 Pac Blue (clone SJ25-C1)	Thermo-Fisher	Cat# MHCD1928; RRID:AB_10373689
CD4 PERCP-Cy5.5 (clone L200)	BD Biosciences	Cat# 552838; RRID:AB_394488
CD8 BV510 (clone RPA-8)	Biolegend	Cat# 301048; RRID:AB_2561942
PD-1 BV711 (clone EH12.2H7)	Biolegend	Cat# 329928; RRID:AB_11218612
CD27 PE-Cy5 (clone 1A4)	Beckman Coulter	Cat# 6607107; RRID:AB_10641617)
CD45RA BV570 (clone HI100)	Biolegend	Cat# 304132; RRID:AB_2563813
CD3 BV650 (clone OKT3)	Biolegend	Cat# 317324; RRID:AB_2563352
IFN-g Alexa 700 (clone B27)	BD Biosciences	Cat# 557995; RRID:AB_396977
TNF BV786 (clone Mab11)	Biolegend	Cat# 502948; RRID:AB_2565858
IL-2 APC (clone MQ1-17H12)	Biolegend	Cat# 500310; RRID:AB_315097
Anti-human IgG (Fab-specific)-horseradish peroxidase	Sigma Aldrich	Cat# A0170; RRID:AB_257868

**Biological samples**		

Convalescent health care worker blood samples	Groote Schuur Hospital	https://www.gsh.co.za

**Chemicals, peptides, and recombinant proteins**		

PepTivator® SARS-CoV-2 Prot_S	Miltenyi Biotech	Cat #130-126-701
PepTivator® SARS-CoV-2 Prot_S1	Miltenyi Biotech	Cat# 130-127-048
SARS-CoV-2 ancestral, Delta and Omicron spike synthetic peptides	TC Peptide Lab	https://tcpeptidelab.com
BTA Nucleocapsid (1mg)	BioTech Africa	Cat# BA25-C
PtX™ SARS-CoV-2 Spike Protein (S1, Rabbit FC)	Cape Bio Pharms (Pty) Ltd	Cat # CB_0002.2
O-phenylenediamine dihydrochloride (OPD), Sigmafast TM	Sigma Aldrich	P9187-50SET
Casein, Hammarsten Bovine	Sigma Aldrich	E0789-500
PBS with 0.05% tween, pH 7.4	Sigma Aldrich	P3563-10PAK

**Critical commercial assays**		

LIVE/DEAD™ Fixable VIVID Stain	Invitrogen	Cat #L34955
Cytofix/Cyto perm buffer	BD Biosciences	Cat # 554722
CellFIX buffer	BD Biosciences	Cat # 340181
Software		
FACS Diva 9	BD Biosciences	https://www.bdbiosciences.com
FlowJo 10	FlowJo, LLC	https://www.flowjo.com
Graphpad Prism 9	Graphpad	https://graphpad.com
BioRender	BioRender	https://biorender.com

### Resource availability

#### Lead contact

Further information and requests for resources and reagents should be directed to and will be fulfilled by the lead contact: Catherine Riou (cr.riou@uct.ac.za).

#### Materials availability

Materials will be made available from the lead contact with a completed Materials Transfer Agreement.

### Experimental model and subject details

#### Human subjects

Participants were recruited from a longitudinal study of healthcare workers (HCW; n = 400) enrolled from Groote Schuur Hospital (Cape Town, Western Cape, South Africa). HCW in this cohort were recruited between July 2020 and January 2021, and vaccination with the first dose Johnson and Johnson Ad26.COV2.S in the Sisonke Phase 3b trial took place between 17 February and 26 March 2021, while the second dose was administered between 10 November and 17 December 2021. Participants were selected for inclusion in this study based on the availability of PBMC and plasma, and who fell into one of four groups: (1) One exposure to SARS-CoV-2 either though a single Ad26.COV2.S vaccination or evidence of previous SARS-CoV-2 infection by diagnostic PCR test or serial serology (n = 73); (2) Two exposures to SARS-CoV-2 through a combination of one or two Ad26.COV2.S vaccinations and/or evidence of SARS-CoV-2 infection by diagnostic PCR test or serial serology (n = 67); (3) Three exposures to SARS-CoV-2 through a combination of one or two Ad26.COV2.S vaccinations and evidence of SARS-CoV-2 infection either prior to and/or after vaccination as confirmed by diagnostic PCR test or serial serology (n = 40); and (4) Four exposures to SARS-CoV-2 comprising two Ad26.COV2.S vaccinations and evidence of SARS-CoV-2 infection both prior to vaccination as well as breakthrough infection post-vaccination confirmed by diagnostic PCR test or serial serology (n = 10). Infections prior to vaccination were classified based on spike or nucleocapsid antibody positivity, and vaccine breakthrough infections were classified as such based on either a positive SARS-CoV-2 PCR test, or the presence of N antibodies either (1) where they did not exist in prior serial samples; or (2) where they were more than 2-fold higher than the previous timepoint. The study was approved by the University of Cape Town Human Research Ethics Committee (HREC 190/2020 and 291/2020). Written informed consent was obtained from all participants.

### Method details

#### Isolation of PBMC

Blood was collected in heparin tubes and processed within 3 h of collection. Peripheral blood mononuclear cells (PBMC) were isolated by density gradient sedimentation using Ficoll-Paque (Amersham Biosciences, Little Chalfont, UK) as per the manufacturer’s instructions and cryopreserved in freezing media consisting of heat-inactivated fetal bovine serum (FBS, Thermofisher Scientific) containing 10% dimethyl sulfoxide (DMSO) and stored in liquid nitrogen until use.

#### SARS-CoV-2 antigens

For serology assays, commercially available recombinant SARS-CoV-2 spike (S1, Cape Bio Pharms, Cape Town, South Africa) and nucleocapsid (BioTech Africa, Cape Town, South Africa) proteins were used. Proteins were reconstituted in PBS (PBS) at a stock concentration of 500 μg/mL and stored at −80°C until use. For T cell assays, we used peptides covering the full-length SARS-CoV-2 spike protein, by combining two commercially available peptide pools of 15mer sequences with 11 amino acids (aa) overlap (PepTivator, Miltenyi Biotech, Bergisch Gladbach, Germany). These peptides are based on the ancestral strain and cover the N-terminal S1 domain of SARS-CoV-2 from aa 1 to 692, as well as the majority of the C-terminal S2 domain. Pools were resuspended in distilled water at a concentration of 50 μg/mL and used at a final concentration of 1 μg/mL. To determine T cell responses to SARS-CoV-2 variants, peptides were synthesized that spanned the entire SARS-CoV-2 spike protein and corresponded to the ancestral sequence (GenBank: MN_908947.3), the Delta SARS-CoV-2 variant (B.1.617.2; GISAID: EPI_ISL_2020950) or to the Omicron SARS-CoV-2 variant BA.1 (B.1.1.529; GISAID: EPI_ISL_6795848) carrying in the spike sequence all the 38 currently described mutations (A67V, H69del, V70del, T95l, G142D, V143del, Y144del, Y145del, S152W, N211del, L212l, ins214EPE, G339D, S371L, S373P, S375F, K417N, N440K, G446S, S477N, T478K, E484A, Q493R, G496S, Q498R, N501Y, Y505H, T547K, D614G, H655Y, N679K, P681H, N764K, D796Y, N856K, Q954H, N969K, L981F). Peptides were 15-mers overlapping by 10 amino acids and were synthesized as crude material (TC Peptide Lab, San Diego, CA). All peptides were individually resuspended in DMSO at a concentration of 10–20 mg/mL. Megapools for each antigen were created by pooling aliquots of these individual peptides in the respective SARS-CoV-2 spike sequences, followed by sequential lyophilization steps, and resuspension in DMSO at 1 mg/mL. Pools were used at a final concentration of 1 μg/mL with an equimolar DMSO concentration in the non-stimulated control.

#### SARS-CoV-2 spike and nucleocapsid ELISA

Two μg/mL of spike or nucleocapsid protein was used to coat 96-well, high-binding plates and incubated overnight at 4°C. Plates were incubated in a blocking buffer consisting of 1% casein, 0.05% Tween 20 in PBS for 1 h. Plasma samples were diluted to 1:50 in dilution buffer (0.5% casein, 0.05% Tween 20 and PBS), added to the plates and incubated at room temperature for 2 h. After washing, anti-human IgG conjugated to horseradish peroxidase (BD Biosciences, San Jose, CA, USA) was diluted to 1:5000 in dilution buffer and added to the plates for 1 h followed by O-phenylenediamine dihydrochloride (OPD) substrate for 12 min (Sigma-Aldrich). Upon stopping the reaction with 3M HCL, absorbance was measured at a wavelength of 490nm.

#### Cell stimulation and flow cytometry staining

Cryopreserved PBMC were thawed, washed and rested in RPMI 1640 containing 10% heat-inactivated FCS for 4 h prior to stimulation. PBMC were seeded in a 96-well V-bottom plate at ∼2 × 10^6^ PBMC per well and stimulated with SARS-CoV-2 spike or nucleocapsid peptide pools. All stimulations were performed in the presence of Brefeldin A (10 μg/mL, Sigma-Aldrich, St Louis, MO, USA) and co-stimulatory antibodies against CD28 (clone 28.2) and CD49days (clone L25) (1 μg/mL each; BD Biosciences, San Jose, CA, USA). As a background control, PBMC were incubated with co-stimulatory antibodies, Brefeldin A and an equimolar amount of DMSO.

After 16 h of stimulation, cells were washed, stained with LIVE/DEAD Fixable VIVID Stain (Invitrogen, Carlsbad, CA, USA) and subsequently surface stained with the following antibodies: CD14 Pac Blue (TuK4, Invitrogen Thermofisher Scientific), CD19 Pac Blue (SJ25-C1, Invitrogen Thermofisher Scientific), CD4 PERCP-Cy5.5 (L200, BD Biosciences, San Jose, CA, USA), CD8 BV510 (RPA-8, Biolegend, San Diego, CA, USA), PD-1 BV711 (EH12.2H7, Biolegend, San Diego, CA, USA), CD27 PE-Cy5 (1A4, Beckman Coulter), CD45RA BV570 (HI100, Biolegend, San Diego, CA, USA). Cells were then fixed and permeabilized using a Cytofix/Cyto perm buffer (BD Biosciences) and stained with CD3 BV650 (OKT3) IFN-ɣ Alexa 700 (B27), TNF-α BV786 (Mab11) and IL-2 APC (MQ1-17H12) from Biolegend. Finally, cells were washed and fixed in CellFix (BD Biosciences). Samples were acquired on a BD LSR-II flow cytometer and analyzed using FlowJo (v10, FlowJo LLC, Ashland, OR, USA). Cells were gated on singlets, CD14^−^ CD19^−^, live lymphocytes and memory cells (excluding naive CD27^+^ CD45RA + population) and a full gating strategy is available in Figure S5. Results are expressed as the frequency of CD4^+^ or CD8^+^ T cells expressing IFN-ɣ, TNF-α or IL-2. Due to high TNF-α backgrounds, cells producing TNF-α alone were excluded from the analysis. Cytokine responses presented are background subtracted values (from the frequency of cytokine produced in unstimulated cells).

### Quantification and statistical analysis

Statistical analyses were performed in Prism (v9.3.1; GraphPad Software Inc, San Diego, CA, USA). Non-parametric tests were used for all comparisons. The Kruskal-Wallis with Dunn’s multiple comparison test or Mann-Whitney test were used for unpaired samples. The Wilcoxon two-tailed test was used for paired samples. All correlations reported are non-parametric Spearman’s correlations. Chi-squared tests were used for comparisons between proportion of responders represented as pie charts. p values less than 0.05 were considered statistically significant. Details of statistical analyses performed for each experiment are described in the figure legends.

## Data Availability

All data reported in this paper will be shared by the lead contact upon request. This paper does not report the original code. Any additional information required to reanalyze the data reported in this paper is available from the lead contact upon request.

## Supplemental information

Document S1. Figures S1–S5 and Tables S1 and S2

Document S2. Article plus supplemental information
