# Supplementary material for: Impact of SARS-CoV-2 exposure history on the T cell and IgG response
Source: Cell Rep Med. 2022 Dec 22;4(1):100898. doi: 10.1016/j.xcrm.2022.100898 (PMC9771741; doi:10.1016/j.xcrm.2022.100898)
Supplement: Document S1. Figures S1–S5 and Tables S1 and S2 [file mmc1.pdf]

Cell Reports Medicine, Volume *4*

Supplemental information

Impact of SARS-CoV-2 exposure history

on the T cell and IgG response

Roanne Keeton, Marius B. Tincho, Akiko Suzuki, Ntombi Benede, Amkele Ngomti, Richard Baguma, Masego V. Chauke, Mathilda Mennen, Sango Skelem, Marguerite Adriaanse, Alba Grifoni, Daniela Weiskopf, Alessandro Sette, Linda-Gail Bekker, Glenda Gray, Ntobeko A.B. Ntusi, Wendy A. Burgers, and Catherine Riou

Supplemental Figure S1

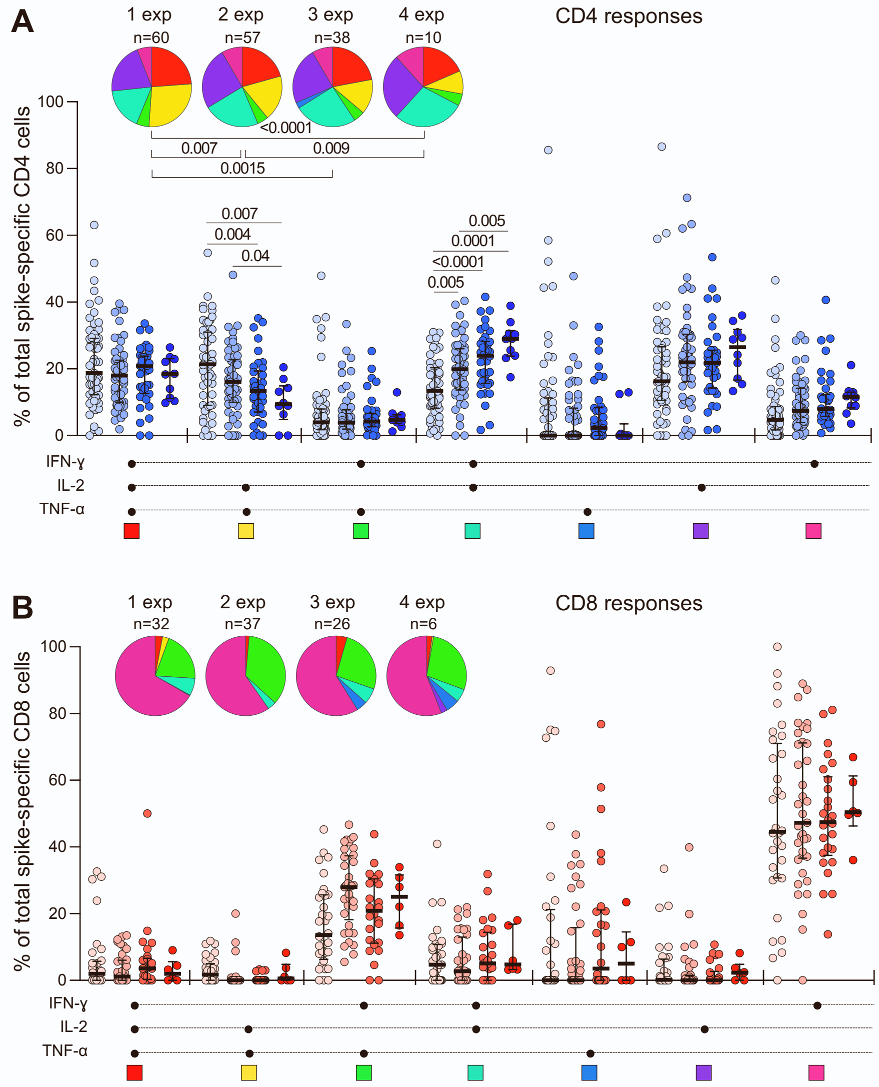

Figure S1: Polyfunctional profiles of spike-specific T cells upon repeated SARS-CoV-2 exposures. Related to Figure 1. (A) Comparison of the polyfunctional profile of spike-specific CD4+ T cells based on the number of SARS-CoV-2 antigen exposures (1 to 4). (B) Comparison of the polyfunctional profile of spike-specific CD8+ T cells. The median proportion and IQR are shown. Each response pattern (i.e., any possible combination of IFN-γ, IL-2 or TNF-α expression) is color-coded, and data are summarized in the pie charts. Statistical comparisons were performed using a permutation test for the pies and a Wilcoxon unpaired t-test for each response pattern. The number of participants included in each graph is indicated on top of the pies.

A

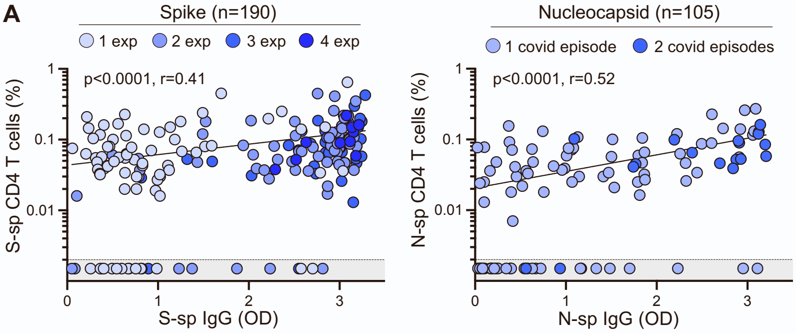

B

		Spike-sp CD4 (%)	Spike-sp CD8 (%)	Spike-sp IgG (OD)
Time since last exposure	1 exposure (n=73)	p = 0.109<br>r = -0.189	p = 0.086<br>r = -0.203	p = 0.084<br>r = 204
	2 exposures (n=54)	p = 0.057<br>r = 0.273	p = 0.144<br>r = 0.201	p = 0.257<br>r = 0.157
	3 exposures (n=27)	p = 0.164<br>r = -0.277	p = 0.582<br>r = 0.111	p = 0.186<br>r = -0.262

Figure S2: Relationship between the frequency of SARS-CoV-2-specific CD4+ T cells with SARS-CoV-2-specific IgG and time since last SARS-CoV-2 antigen exposure. Related to Figure 1. (A) Correlation between the frequency of spike-specific CD4+ T cells and the magnitude of spike-specific IgG (left panel) and correlation between the frequency of nucleocapsid-specific CD4+ T cells and the magnitude of nucleocapsid-specific IgG (right panel). (B) Correlation between the magnitude of spike-specific CD4+ T cells, spike-specific CD8+ T cells or spike-specific IgG and the time since last SARS-CoV-2 antigen exposure in each study group. Correlations were tested by a two-tailed non-parametric Spearman rank test.

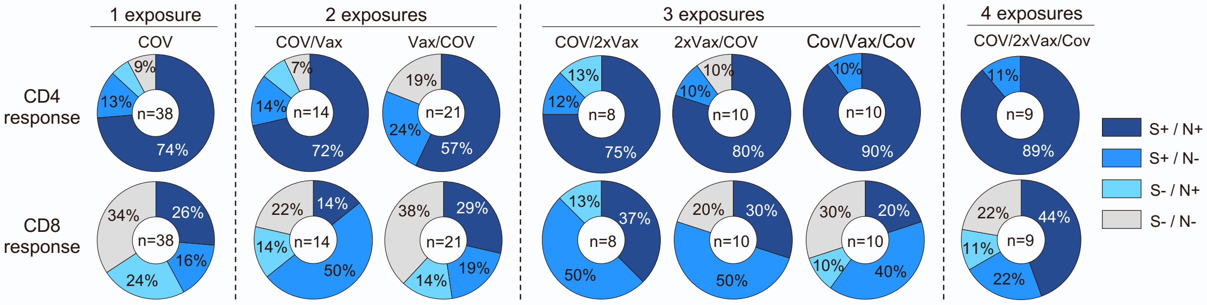

Figure S3: Distribution of the proportion of spike and/or nucleocapsid CD4 (top) and CD8 (bottom) responders upon different infection/vaccination exposures. Related to Figure 2. The order of infection/vaccination is indicated at the top of the pie charts. The number of participants in each sub-group is indicated inside each pie chart. S: spike, N: nucleocapsid.

Supplemental Figure S4

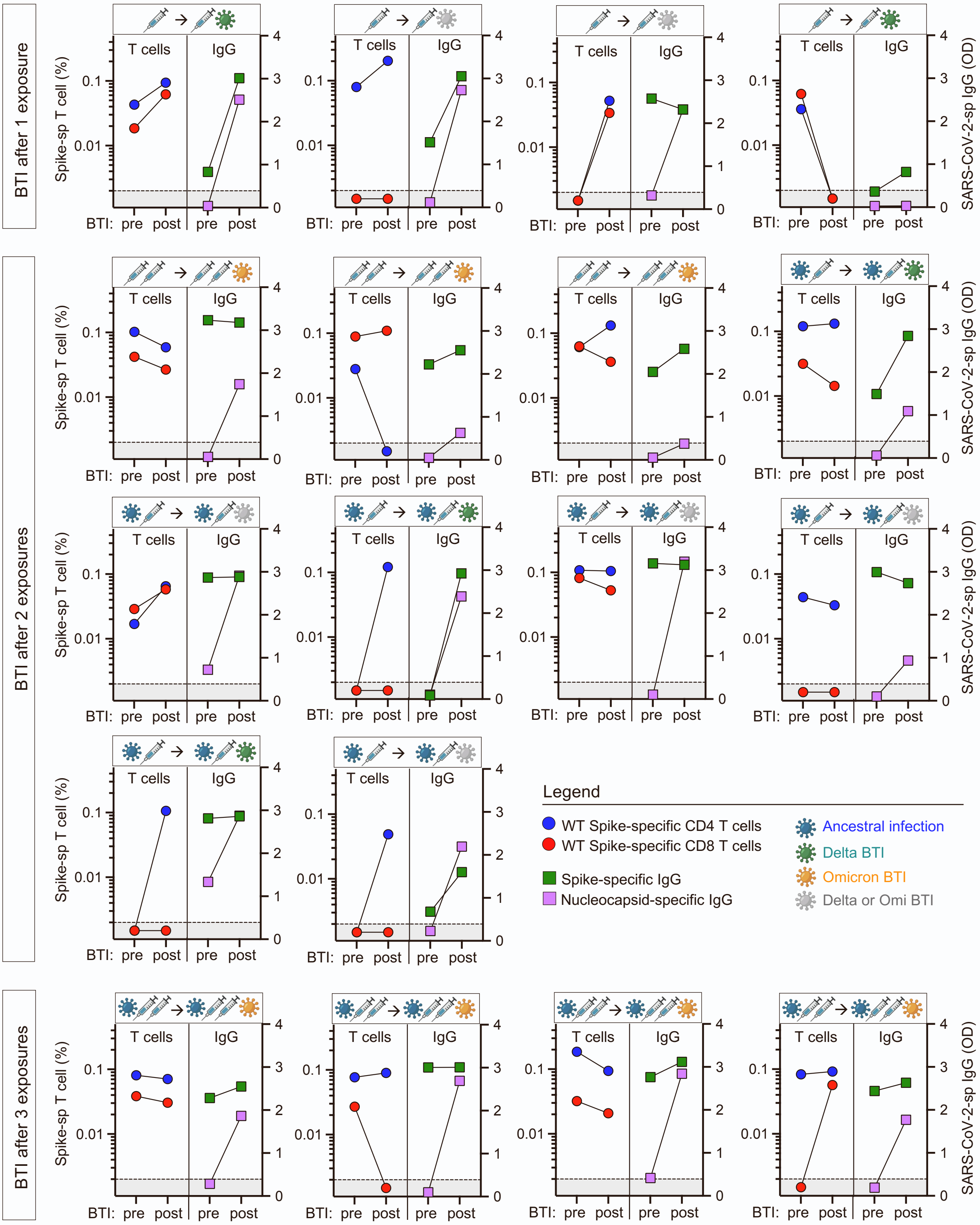

Figure S4: Immunological profiles of breakthrough infections after 1, 2 or 3 exposures. Related to Figure 5. Individual profiles pre- and post-BTI infection of the frequency of ancestral spike CD4+ (blue circles) and CD8+ (red circles) T cell responses (left axis) or the magnitude of IgG responses to spike (green squares) or nucleocapsid (purple squares) on the right axis. The order of exposure (vaccination or infection) for each individual is indicated above the graph. Each SARS-CoV-2 infecting variant is depicted with a different color (see key).

Supplemental Figure S5

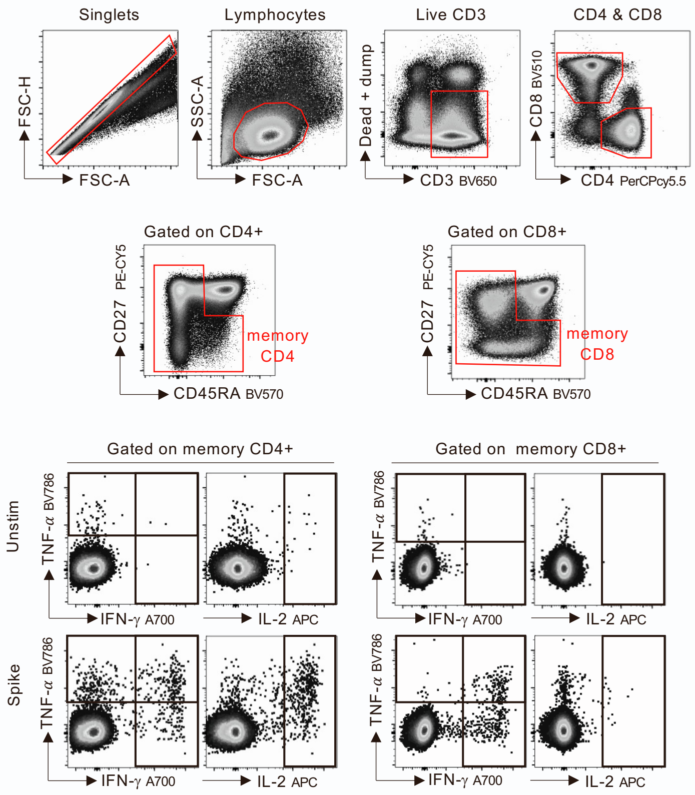

Figure S5: Flow cytometry gating strategy. Related to STAR methods. Gating strategy and representative examples of SARS-CoV-2 spike-specific IFN-γ, IL-2 and TNF-α production in CD4+ and CD8+ T cells.

Table S1: **Clinical characteristics of participants grouped based on the order of SARS-CoV-2 antigen exposures. Related to Fig. 2.**

	1 exp	1 exp	2 exp	2 exp	2 exp	3 exp	3 exp	3 exp	4 exp
	Vax	COV	Vax/Vax	COV/Vax	Vax/COV	COV/Vax/Vax	Vax/Vax/COV	COV/Vax/COV	
N	33	40	9	36	22	20	10	10	10
Age (median, IQR)	47 [35-56]	35 [29-42]	36 [28-58]	38 [29-51]	39 [30-50]	35 [30-43]	49 [33-54]	43 [30-54]	37 [27-50]
Gender (% female)	69.7%	62.5%	66.7%	72.2%	66.7%	60%	80%	90%	80%
Time after last exposure, months<br>IQR<br>n with data available	4.8<br>[1.3-6.03]<br>n=33/33	5.36<br>[1.73-6.74]<br>n=40/40	0.76<br>[0.71-0.82]<br>n=9/9	5.23<br>[4.76-6.08]<br>n=36/36	1.05<br>[0.79-1.81]<br>n=9/22	0.79<br>[0.69-1.09]<br>n=20/20	1.02<br>[0.99-1.38]<br>n=3/10	1.28<br>[0.58-1.94]<br>n=4/10	0.69<br>[0.69-0.99]<br>n=3/10
With COVID-19 infection	0%	100%	0%	100%	100%	100%	100%	100%	100%

Exp: exposure, Vax: SARS-CoV-2 vaccination, COV: COVID-19 episode, IQR: Interquartile range.

Table S2: Clinical characteristics of breakthrough infections with longitudinal data. Related to Fig. 4.

				Pre-BTI							Post-BTI								
PID	BTI Strain	Age	Gender	Nb Expo	Sequence of Exposures	Time since last expo (Month)	S-CD4 (%)	S-CD8 (%)	S Abs	N Abs	Nb Expo	BTI diagnostic (PCR or N abs)	Sequence of Exposures	Time since last expo (Month)	S-CD4 (%)	S-CD8 (%)	S Abs	N Abs
1201	Unk	41	Female	2	Pre/Vax	6.34	0.0170	0.0290	2.87	0.72	3	N abs	Pre/Vax/BTI	<5M	0.0650	0.0582	2.88	2.91
1314	Unk	51	Female	1	Vax	2.63	0.0800	0.0015	3.05	0.12	2	N abs	Vax/BTI	<6.5M	0.2030	0.0015	3.05	2.73
1343	Unk	27	Female	2	Pre/Vax	3.85	0.0015	0.0015	0.68	0.23	3	N abs	Pre/Vax/BTI	Unk	0.0490	0.0015	1.60	2.19
1381	Unk	29	Female	1	Vax	3.85	0.0015	0.0015	2.58	0.32	3	N abs	Pre/Vax/BTI	<6.5M	0.1050	0.0527	3.12	3.20
1357	Unk	53	Female	2	Pre/Vax	5.06	0.1080	0.0820	3.16	0.10	3	N abs	Pre/Vax/BTI	<6M	0.0330	0.0015	2.74	0.94
1389	Unk	56	Female	2	Pre/Vax	5.13	0.0440	0.0015	3.00	0.10	2	N abs	Vax/BTI	<6.5M	0.0523	0.0340	2.32	2.32
1100	Omi	22	Female	3	Pre/Vax/Vax	0.69	0.1020	0.0422	3.23	0.05	4	N abs	Pre/Vax/Vax/BTI	<2M	0.0590	0.0267	3.18	1.74
1134	Omi	25	Male	3	Pre/Vax/Vax	0.79	0.0770	0.0270	3.01	0.10	4	N abs	Pre/Vax/Vax/BTI	<2M	0.0900	0.0015	3.01	2.70
1310	Omi	48	Female	3	Pre/Vax/Vax	0.72	0.1860	0.0320	2.76	0.416	4	N abs	Pre/Vax/Vax/BTI	<2.5 M	0.0940	0.0208	3.12	2.84
1313	Omi	57	Female	2	Vax/Vax	0.69	0.0610	0.0626	2.05	0.05	3	N abs	Vax/Vax/BTI	<2.5 M	0.1320	0.0362	2.58	0.38
1085	Omi	39	Male	2	Vax/Vax	0.72	0.0280	0.0890	2.22	0.05	3	N abs	Vax/Vax/BTI	<2M	0.0015	0.1090	2.55	0.63
1043	Omi	25	Male	2	Vax/Vax	1.08	0.0810	0.0384	2.28	0.28	3	N abs	Vax/Vax/BTI	<1.5M	0.0710	0.0304	2.55	1.86
1060	Omi	38	Female	3	Pre/Vax/Vax	1.48	0.0830	0.0015	2.44	0.19	4	N abs	Pre/Vax/Vax/BTI	<1.5M	0.0920	0.0568	2.63	1.77
1033	Delta	31	Male	2	Pre/Vax	0.92	0.1200	0.0316	1.49	0.06	3	PCR	Pre/Vax/BTI	2.04	0.1320	0.0144	2.84	1.09
1203	Delta	56	Female	2	Pre/Vax	0.92	0.0015	0.0015	0.09	0.10	3	PCR	Pre/Vax/BTI	0.92	0.1210	0.0015	2.93	2.39
1399	Delta	28	Female	2	Pre/Vax	0.89	0.0015	0.0015	2.82	1.34	3	PCR	Pre/Vax/BTI	0.46	0.1060	0.0015	2.86	2.88
1028	Delta	47	Female	1	Vax	0.79	0.0430	0.0186	0.83	0.04	2	N abs	Vax/BTI	<4M	0.0940	0.0620	3.01	2.51
1104	Delta	34	Female	1	Vax	0.95	0.0360	0.0620	0.37	0.03	2	PCR	Vax/BTI	1.87	0.0015	0.0015	0.82	0.03

PID: patient identification number, Pre: Prior COVID-19 infection, Vax: SARS-CoV-2 vaccination, BTI: breakthrough infection, Nb expo: number of exposures, S-CD4: Spike-specific CD4 T cells, S-CD8: Spike-specific CD8 T cells, S abs: Spike-specific IgG, N abs: Nucleocapsid-specific IgG. Unk: unknown, Omi: Omicron.
BTI diagnostic was ascertained by a positive SARS-CoV-2 PCR test (PCR), conversion to nucleocapsid seropositivity (N abs) or a >2-fold increase in anti-N IgG optical density (OD value..
